# Unmet Need for Family Planning Among Women Attending Primary Healthcare in Alahsa, Saudi Arabia

**DOI:** 10.7759/cureus.62525

**Published:** 2024-06-17

**Authors:** Narjes A Alramadhan, Maria M Busaleh, Hasan M Alhaddad

**Affiliations:** 1 Family Medicine, Ministry of Health, Alahsa, SAU

**Keywords:** unmet needs, saudi arabia, primary care, family planning, contraception

## Abstract

Backgrounds: Enhancing maternal health quality is a concern among researchers globally. According to the World Health Organization (WHO), one factor in women’s health and empowerment is the rate of use of various contraceptive techniques. The WHO defines unmet contraceptive need as the discrepancy between a woman’s desire to delay or cease childbearing and lack of contraception use to achieve this goal. Our study was designed to measure the unmet need for family planning and contraceptive use among married Saudi women attending primary healthcare centers in Alahsa, Saudi Arabia.

Methodology: A cross-sectional study was carried out using multistage cluster stratified sampling. The study included all married Saudi women aged 18-49 attending primary health centers. A structured questionnaire from the United States Agency for International Development Demographic and Health Surveys Methodology was used. Data analysis was performed using the statistical software IBM SPSS version 29 (IBM Corp., Armonk, NY).

Results: In all, 430 individuals were included. The participants’ ages ranged from 19 to 49 years (33.4 ± 7 years). Among them, 50 (11.6%) were pregnant. Among those who were not pregnant, 268 (62.3%) were using a method of contraception. Based on the definitions adopted in this study, 90 (20.9%) had unmet needs for family planning, and 340 (79.1%) had their needs met. The total demand for family planning was estimated to be 83.2%. The percentage of demand for family planning satisfied by a modern contraceptive method was 46.9%.

Conclusion: Although Alahsa has a lower unmet need rate (20%) than other cities in Saudi Arabia, it remains notably higher than the average rate in Northern African and Western Asian countries, which is 10.9%. A number of factors, including nulliparity and having more than two children, were associated with unmet contraceptive needs. The majority of women who did not use contraceptives had concerns about the side effects and inconvenience of use.

## Introduction

Enhancing maternal health quality is a concern among researchers globally. According to the World Health Organization (WHO), one of the factors in women’s health and empowerment is the rate of use of various contraceptive techniques [1]. Family planning is a voluntary approach in which individuals either restrict or space the number of children born, which is accomplished through the use of contraceptive methods [2]. According to the Saudi Household Health Survey, the prevalence of modern contraceptive use was 30.4% in 2018 [3]. Despite the growing prevalence of contraceptive use in Saudi Arabia, its demand remains unmet. Over the past decade, the percentage of unplanned pregnancies in Middle Eastern and North African countries has increased from 15% to 58%, suggesting inappropriate use of contraceptives or an unmet contraceptive need [4]. The WHO defines unmet contraceptive need as the discrepancy between a woman’s desire to delay or cease childbearing and lack of contraception use to achieve this goal [5]. According to the United Nations Department of Economic and Economic Affairs, the proportion of women aged 15-49 who have their need for family planning satisfied by modern contraceptive methods in Saudi Arabia was 40.1% in 2000 and 43.6% in 2020, compared to 55% in 2000 and 62.9% in 2020 for other Northern Africa and Western Asia countries and 72.8% in 2000 and 79.5% in 2020 in other developed countries [6]. A study conducted by Khalil et al. in Abha region, Saudi Arabia, in 2018 found that 32.6% of women had an unmet need for family planning services, where the unmet needs were mainly due to lack of access to family planning methods, insufficient knowledge, religious prohibition, fear of adverse side effects from previous contraceptives, and spousal opposition, respectively [4]. In Saudi Arabia, several contraceptives are available as over-the-counter medication, where pharmacist advice may be the only source of information. As a consequence of self-medication, pills are likely to be used in an inappropriate manner, which can lead to poor efficacy and unintended pregnancy [7]. However, women in Saudi Arabia tend to use self-prescribed contraception due to less access to healthcare services for women, and a large number of children [8].

In Saudi Arabia, research on the unmet need for family planning is limited, and little is known about the reason for not getting contraceptive counseling. Thus, this study aimed to measure the unmet need and demand for family planning among Saudi women attending primary healthcare centers (PHCCs) in Alahsa, Saudi Arabia.

## Materials and methods

Study setting and study population

A cross-sectional study was carried out in the Alahsa region of Saudi Arabia. According to the estimated census of 2017 from the General Authority of Statistics, the Alahsa region has 416,844 Saudi women [9]. The present study was conducted in the PHCCs of the Alahsa region. Data collection was performed between July and November 2023.

Sample size calculation

Sample size estimation was done using $n = (Z^2 * P * (1-P)) / d^2$, where n is the sample, Z is the statistic corresponding to the level of confidence, P is the expected prevalence, and d is precision​​​​​​​.

Due to the non-availability of data on this subject in Saudi Arabia, the prevalence rate of unmet needs was 50%, at a 95% confidence level, z score of 1.96, and margin of error of 5%. A sample size of 384 women was calculated.

Sampling technique

Multistaged cluster stratified sampling was used. Our target population was selected from the Alahsa region. The region was sub-grouped into four zones according to the official allotment of the Alahsa Health Cluster (Northern, Middle, Eastern, and Southern) as the primary sampling units. In the second stage, all PHCCs within those zones were listed, and randomization software was used to obtain three PHCCs from each zone as our secondary sampling units. In the third stage, a convenience sample from the randomly selected PHCCS was used as the ultimate sampling unit. The sample was equally distributed among the chosen PHCCs.

Eligibility criteria

All married Saudi women aged 18-49 attending the PHCCs who had not reached menopause were included in the study. Women not fulfilling the above criteria were excluded.

Data collection tool and technique

A structured questionnaire was conducted using a United States Agency for International Development (USAID) demographic and health survey methodology (household, women’s, and men’s DHS model questionnaire - phase 6 (2008-2013) was used) [10]. The questionnaire contained two sections; The first section included sociodemographic data. The second section included questions structured to define the participant into two primary categories using filter questions to reflect the flow of the questions. Group 1 consisted of women whose contraceptive needs had been met, while Group 2 consisted of women who had unmet contraceptive needs. The questionnaire also contained a question regarding the source of contraceptive information and a question to explore the reason for not using contraceptives for the unmet need group. The data were collected through face-to-face interviews with women attending the PHCCs; the interview was done in a closed room to ensure the participants’ privacy.

Data analysis

After data were extracted, they were revised, coded, and fed to the statistical software IBM SPSS version 29 (IBM Corp., Armonk, NY). All statistical analysis was done using two-tailed tests. Significance was adopted at p < 0.05 for interpretation of the results of tests. The collected participants’ data are reported as percentages for categorical variables and mean and standard deviation for numerical variables. Chi-square was used to test the association between categorical variables.

## Results

Baseline information of the participants

Table 1 illustrates the demographic characteristics of the participants. The study included 430 participants who fulfilled the inclusion criteria. The participants’ ages ranged between 19 and 49 years, with a mean of 33.4 ± 7 years. The duration of marriages in the study sample ranged from zero to 33 years, with a mean of 11.8 ± 7.6 years. The majority of the respondents had an undergraduate or graduate degree 306 (71.1%), were housewives 242 (56.3%), had more than two children 238 (55.3%), and had an income level of 5,000-10,000 rials per month 188 (43.7%).

**Table 1:** Sociodemographic characteristics of the study participants

Variable		N	Percent
Age range of the woman	18-29 years old	141	32.8
	30-39 years old	194	45.1
	40-49 years old	95	22.1
Residency within Alahsa province	The Northern zone	118	27.4
	The central zone	104	24.2
	The southern zone	104	24.2
	The eastern zone	104	24.2
Educational level of the wife	Illiterate	2	0.5
	Primary school	8	1.9
	Middle school	18	4.2
	Secondary school	93	21.6
	Diploma	34	7.9
	Bachelor degree	240	55.8
	Higher education	32	7.4
Educational level of the husband	Primary school	14	3.3
	Middle school	39	9.1
	Secondary school	118	27.4
	Diploma	77	17.9
	Bachelor degree	158	36.7
	Higher education	24	5.6
Occupation of the wife	Housewife	242	56.3
	Student	26	6
	Working	159	37
	Businesswomen	3	0.7
Occupation of the husband	Unemployed	11	2.6
	Working	365	84.9
	Businessman	21	4.95
	Freelance job	9	2.1
	Retired	24	5.6
Family income	Less than 5000 rials per month	85	19.85
	5000 – 10000 rials per month	188	43.7
	More than 5000 rials per month	157	36.5
Number of children	0	35	8.1
	1	70	16.3
	2	87	20.2
	More than 2	238	55.3
Currently pregnant	Yes	50	11.6
	No	373	86.7
	Don't know	7	1.6

Demand for family planning

Out of 430 participants, 50 (11.6%) were pregnant. Among those not pregnant, 268 (62.3%) used either modern or traditional contraceptives. Of these, 168 (62.7%) used a modern contraceptive, and 146 (54.5%) used a traditional one. Out of the traditional method users, 44 (30.1%) perceived themselves as not using a contraceptive, and 102 (69.9%) perceived themselves as a contraceptive user. Table 2 illustrates the family planning methods used by the participants.

**Table 2:** Family planning methods utilized by the participants The responses were gathered through multiple-choice questions.

Contraceptive method	Responses	Percent
Withdrawal	106	31.70
Condom	71	21.30
Contraceptive pills	54	16.20
Intrauterine device	35	10.50
Rhythm method	35	10.50
Implant	22	6.60
Tubal ligation/vasectomy	7	2.10
Injectable contraceptive	3	0.90
Abstinence	1	0.30
Total	334	100.00

**Figure 1:**
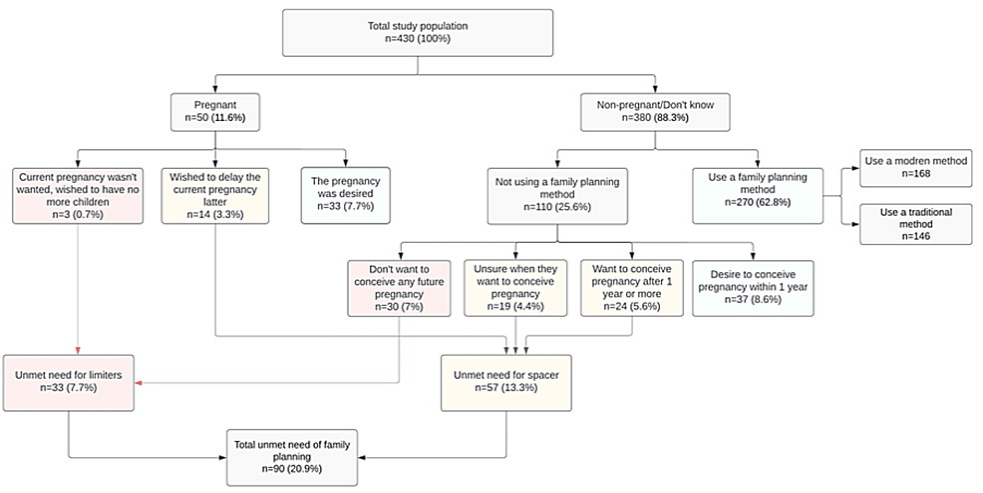
Unmet need and demand for family planning Image Credits: Narjes Alramadhan

Based on the definitions adopted in this study, 90 (20.9%) participants had unmet needs for family planning, and 340 (79.1%) had their needs met. To estimate the total demand for family planning, we need to calculate the sum of unmet needs and the current rate of contraceptive use in any form [10]. In our study, this value was estimated to be 358 (83.2%). From this data, we can calculate the percentage of demand for family planning that has been satisfied by a modern contraceptive method. This was achieved by dividing the number of people using modern contraceptives by the total demand [10], which in our study was found to be 46.9%. Figure 1 illustrates the unmet need and family planning demand.

Association between sociodemographic variables with unmet need for family planning

A significant statistical association was found between the number of children a participant had and their unmet need for family planning: (3, N = 430) = 10.3, p-value = 0.016. Participants who had one or two children were more likely to have their needs met compared to those who had no children or more than two children. There was no statistically significant association between unmet needs for family planning and age groups, marriage duration, zone of residence, education of the husband, occupation of wife and husband, or income level (p-value> 0.05). Table 3 illustrates the significant associations between family demand and various sociodemographic factors.

**Table 3:** Significant associations between family demand and sociodemographical factors

Variable		Unmet need	Met the need	X^2^	P-value	df
Number of children	0	11	24	10.31	0.016	3
	1	7	63			
	2	14	73			
	More than 2	58	180			

The association between unmet needs and the wife’s educational level could not be performed as 25% of the cells had an expected count of less than 5. Analysis of the components of unmet needs revealed a significant association between spacing/limiting needs and various sociodemographic factors. Younger age groups had significantly higher spacing needs, and older age groups had more limiting needs: (2, N = 90) = 35, p ≤ 0.001. Participants with shorter marriage durations had higher spacing needs, while those with longer marriage durations had higher limiting needs: (2, N = 90) = 24.6, p-value = <0.001. There was no statistically significant association between spacing/limiting needs and zone of residence or income level (p-value> 0.05). Table 4 illustrates the significant associations between components of unmet need and sociodemographical factors.

**Table 4:** Significant associations between components of unmet need and sociodemographical factors

Variable		Unmet need for spacer	Unmet need for limiter	X^2^	P-value	df
Age range	18-29 years old	25	0	34.98	<0.001>	2
	30-39 years old	27	13			
	40-49 years old	5	20			
Marriage duration	less than 5 years	15	1	24.64	<0.001>	2
	between 5-10 years	26	5			
	more than 10 years	16	27			

The association between spacing/limiting needs, education levels of the wife and husband, occupation of the wife and husband, and number of children could not be performed as >20% of the cells had an expected count of less than 5.

Source of information of the participants regarding contraceptive methods

Friends and relatives (213 (31.3%)) were the most common sources of information on contraceptive methods, while books and magazines were the least common sources (39 (5.7%)). Interestingly, eight (1.5%) participants reported never having heard about contraceptive methods. Table 5 illustrates the participants’ source of information regarding contraceptive methods.

**Table 5:** Source of information of the participants regarding contraceptive methods Every participant had the chance to choose more than one source of information.

	Responses	Percent
Relatives-friends	213	31.20
Internet	185	27.10
Physician in the privet hospital.	126	18.50
Physician in the phc/hospital	111	16.30
Books/magazines	39	5.70
I didn’t hear about contraceptives before	8	1.20
Total	682	100.00

Reasons for the non-use of family planning methods among women with unmet needs

Among participants with unmet needs, concerns regarding side effects or health 100 (52.6%) were the most common response, followed by inconvenience 35 (18.4%) and husband opposition 19 (10%). The participants’ responses regarding reasons for the non-use of family planning methods are listed in Table 6.

**Table 6:** Reasons for not using family planning methods in participants with unmet needs Every participant in the unmet need group was allowed to select more than one reason for not using a family planning method.

	Responses	Percent
Side effects/health concerns	100	52.60
Inconvenient to use	35	18.40
Husband opposed	19	10.00
Costs too much	10	5.30
Preferred method not available/no method available	7	3.70
I have no source to get contraceptives	7	3.70
I don’t have the knowledge about methods of contraception	6	3.20
I don’t have access to contraceptive counseling/ too far	4	2.10
Religious prohibition	2	1.10
Total	190	100.00

## Discussion

Family planning has become one of the most widely discussed subjects throughout the world. The International Conference on Population and Development and the United Nations Millennium Development Goals have acknowledged family planning as essential to women’s health and that of their children [11,12]. The current study was conducted to assess the unmet need for family planning and contraception usage among married Saudi women who visit PHCCs in Alahsa, Saudi Arabia.

The unmet need for family planning has been defined as the proportion of women who have no desire to become pregnant but do not use any method of contraception [11]. In the conducted study, which included 430 participants, it was found that 20.9% experienced unmet needs in family planning, a rate lower than has been reported in other regions of Saudi Arabia. Conversely, 79.1% of participants had their needs met regarding family planning. A study conducted by Alselmi in Taif City, Saudi Arabia, in 2023 revealed a higher prevalence of unmet need for contraceptive use (49.9%), while a study conducted by Khalil et al. in the Abha region, Saudi Arabia, in 2018 reported a rate of 32.6% for unmet need. This might be explained by the higher number of women in our study sample who were either working or pursuing further education (43.7%), as compared to previous studies conducted in Abha (39%) and Taif City (32.5%) [4,13], as expanding education and employment opportunities for women is a known contributing factor for child spacing.

Studies on the unmet need for family planning have been conducted in many other parts of the world. In Jordan and Egypt, 14% and 11.2% prevalence rates were reported in 2018, respectively [14,15]. According to the United Nations Department of Economic and Social Affairs, Northern African and Western Asian nations have a 10.9% unmet need for contraception [16]. A study from Zambia reported that in 2020, 20% of contraception requirements were unmet [17]. Similarly, in Ethiopia, the prevalence of unmet need rate was determined to be 16.2% in 2019 [18]. These countries’ lower rates of unmet need can be attributed to the establishment of a well-structured national family planning program, such as in Egypt and Zambia, where the unmet need rate was 22.9% in Egypt and 30% in Zambia in 1992 [10].

Among those with unmet needs, 36.7% need limiting, while 63.3% need spacing. This distribution mirrors findings from the Abha study, where 34.4% reported unmet needs for limiting and 65.7% for spacing. Similarly, the Taif study reported unmet needs for limiting and spacing at 9% and 82%, respectively [4,13]. The integration of family planning counseling into standard postnatal care in our region is currently inadequate. Our study emphasizes the necessity of providing immediate counseling on contraception and family planning after childbirth, highlighting the need to include this in routine postnatal care practices. This classification of unmet needs provides valuable insights for program managers, facilitating the selection of appropriate methods to address the diverse contraceptive needs of potential users.

Although the rate of meeting the need for contraception and family planning was high in our study population (79.1%), the proportion of demand satisfied by modern types of contraception was relatively low (46.9%). The most common form of contraception in our study population was withdrawal (31.7%), followed by condoms (21.3%), with abstinence being the least common (0.3%). This finding is crucial as it highlights a significant gap in the utilization of modern contraceptive methods, which are generally more effective in preventing unwanted pregnancies compared to traditional methods. Traditional methods, such as withdrawal and periodic abstinence, have higher failure rates, which can lead to an increased incidence of unintended pregnancies and subsequently, a higher unmet need for family planning. Unintended pregnancies can have profound implications for women, including adverse health outcomes, economic burdens, and social challenges. For instance, women with unintended pregnancies may face higher risks of maternal complications, may be less likely to seek timely prenatal care, and might experience greater emotional and financial stress. By promoting the use of modern contraceptive methods, family planning programs can more effectively meet the needs of women, reduce the rate of unintended pregnancies, and improve overall reproductive health outcomes.

Among the various reasons for not using contraceptives in our study, the top reasons were worries about side effects (52.6%), followed by finding them inconvenient to use (18.4%), and husband’s refusal (10%). In all, 5.3% reported not using contraceptives due to not having knowledge regarding methods of contraceptives or not having access to contraceptive counseling. In the study carried out in Taif, the major reasons for unmet contraception needs were health problems among women (15.6%), husband’s refusal (12.6%), and lack of knowledge (4.6%) [13]. In a study conducted in Abha region, the reasons were lack of access to family planning methods, lack of knowledge of family planning methods, and past experience of side effects [4]. Interestingly, reasons for not using contraceptives are similar in other countries as well, such as in the study conducted by El-Masry et al. in Egypt in 2018 where the most common reasons reported were infrequent sex (27.3%) and fear of side effects (25%), followed by opposition from the husband (15.9%) and perception of sub-fertility (11.4%) [15]. It is worth noting that 44 of the 146 participants in our survey who used traditional methods of contraception described themselves as non-contraceptive users. This might be because these approaches have a high failure rate or are incorrectly used. This emphasizes the need to provide instructional materials and counseling on the correct use of each contraceptive technique.

Additionally, the present study identified significant associations between family demand and various sociodemographic factors, including being nulliparous or having more than two children. This agrees with the study that was conducted at Taif city, which showed that nulliparous women were more likely to have unmet contraception needs [13]. Moreover, our research revealed that younger women and those with shorter marriage durations tended to have unmet needs for spacing. In comparison, older women and those with longer marriage durations were more likely to have unmet needs for limiting. This finding aligns with the study conducted in Abha, Saudi Arabia [4]. Other factors identified in the Abha and Taif studies included low educational level, women who got married or had their first pregnancy after the age of 25, who had only one previous pregnancy, or whose husbands were illiterate, worked as businessmen or were retired were more likely to report unmet contravention needs than their counterparts [4,13].

Unfortunately, our participants’ primary sources of contraceptive information were friends and family, followed by the Internet, and then physicians at private or government PHCCs/hospitals. A minority of participants (1.2%) stated that they had never received information about contraceptive methods. It is essential to recognize that women encounter challenges when it comes to pregnancy prevention; this could be attributed to Saudi Arabia's conservative culture, where such topics are considered personal and not typically discussed with healthcare providers. Additionally, family members and friends being the primary source of information regarding contraceptives for the majority of our sample have a significant influence on the type of contraceptive used since the choice of contraception is influenced by its popularity in society and the misconceptions that circulate among members regarding other methods. Furthermore, besides societal perspectives, this topic faces neglect from spouses and health workers. As a result, women who want to avoid pregnancy find it difficult to do so mainly due to a lack of knowledge; either they do not have information about how to reach contraceptive clinics, or they do not know how to use them.

We must acknowledge some limitations in our study. Firstly, our participants were exclusively drawn from a single city in the kingdom, which may limit the broader applicability of our findings to all Saudi women. Additionally, husbands were not included in the survey despite their potentially significant role in the unmet needs of family planning in our conservative community. Furthermore, Interactions between researchers and participants during data collection could have unintentionally influenced responses, particularly on sensitive topics like contraception. Moreover, the conservative cultural context of Saudi society may have led participants to provide socially desirable answers rather than reflecting their true behaviors and attitudes. Lastly, it is essential to recognize that our study design was cross-sectional. Cross-sectional studies typically do not establish causal associations, limiting our ability to draw definitive conclusions about the relationships observed.

## Conclusions

Although a majority of participants have their family planning needs met, there is a significant portion with unmet needs, particularly due to concerns about side effects and lack of proper knowledge about contraceptives. Efforts are needed to enhance education, access to modern contraceptive methods, engage espouses in family planning counseling sessions, and utilize healthcare providers as key sources of reliable contraceptive information. Our recommendation to improve family planning services and support for individuals with unmet needs is to implement structured programs that provide comprehensive education and convenient access to a variety of contraceptive methods.

## Appendices

The following definitions were adopted in the survey:

Participants who met the need for family planning are defined as follows

Met the need for a spacer:

Women who use any contraceptive method and desire to have children later or are not sure

Met the need for a limiter:

Women who use any contraceptive and have the desire to have no more children

Met the need for family planning:

Woman who desires to get pregnant within one year or less, or a pregnant woman in whom the pregnancy was desired

Participants who have an unmet need for family planning are defined as follows

Unmet need for a spacer:

Woman who is currently pregnant and who wanted to delay this current pregnancy, or woman who is not pregnant and does not use contraceptive and desires to delay pregnancy after one year or more or is not sure about the timing.

Unmet need for limiters:

Woman who is currently pregnant who had wanted no more children, or woman who is not pregnant and does not use contraceptive and desire to have no more children

The total demand for family planning is estimated as the sum of unmet needs and the current prevalence rate of contraceptive use.
